# Preparation of lyophilized recombinant prion protein for TSE diagnosis by RT-QuIC

**DOI:** 10.1186/s13104-018-3982-5

**Published:** 2018-12-14

**Authors:** Soyoun Hwang, Trudy Tatum, Semakaleng Lebepe-Mazur, Eric M. Nicholson

**Affiliations:** Virus and Prion Research Unit, National Animal Disease Center, United States Department of Agriculture, Agricultural Research Service, Ames, IA 50010 USA

**Keywords:** PrP, Scrapie, TSE, Transmissible spongiform encephalopathy, RT-QuIC, Lyophilize, Real time quaking induced conversion

## Abstract

**Objective:**

Transmissible spongiform encephalopathies (TSEs) are a group of fatal neurodegenerative diseases, often referred as prion diseases. TSEs result from the misfolding of the cellular prion protein (PrP^C^) into a pathogenic form (PrP^Sc^) that accumulates in the brain and lymphatic tissue. Amplification based assays such as real-time quaking induced conversion allow us to assess the conversion of PrP^C^ to PrP^Sc^. Real-time quaking induced conversion (RT-QuIC) can be used for the detection of PrP^Sc^ in a variety of biological tissues from humans and animals. However, RT-QuIC requires a continuous supply of freshly purified prion protein and this necessity is not sustainable in a diagnostic laboratory setting.

**Results:**

In this study, we developed a method to dry and preserve the prion protein for long term storage allowing for production of the protein and storage for extended time prior to use and room temperature shipping to appropriate diagnostic laboratory destinations facilitating widespread use of RT-QuIC as a diagnostic method.

## Introduction

Prion diseases are a group of fatal neurologic diseases that result from the misfolding of the monomeric, cellular prion protein (PrP^C^) into an oligomeric, pathogenic form (PrP^Sc^). These diseases are also referred to as transmissible spongiform encephalopathies (TSEs) and include bovine spongiform encephalopathy (BSE) in cattle, scrapie in sheep and goat, chronic wasting disease (CWD) in deer and elk, and Creutzfeldt–Jakob disease (CJD), fatal familial insomnia, Gerstmann–Sträussler–Scheinker syndrome, and kuru in humans. PrP^Sc^ accumulates in the central nervous system in all TSEs, and in cases of scrapie in sheep and CWD in cervids, PrP^Sc^ also accumulates in the lymphoid tissues [1–3].

At present, approved TSE diagnostic tests are all based upon direct detection of PrP^Sc^ using the antibody based approaches immunoblot, enzyme immunoassay (EIA or ELISA), and immunohistochemistry [4, 5]. However, new prion detection tools such as protein misfolding cyclic amplification (PMCA) [6–8] and the real-time quaking-induced conversion (RT-QuIC) assay have gained notoriety in TSE diagnostics based on the high sensitivity afforded by in vitro amplification of PrP^Sc^ [9–11]. While PMCA and RT-QuIC differ in the substrate utilized and the approach to enhance the in vitro conversion, both report on the presence of PrP^Sc^ in a sample through conversion where PrP^Sc^ present in the diagnostic sample acts as a seed to initiate conversion of the provided substrate from a monomeric prion protein to an oligomeric form. RT-QuIC uses bacterially expressed, purified recombinant prion protein (rPrP) and controlled shaking. A steady supply of high quality purified rPrP is required. This is generally accomplished by utilizing one of the proven methods of expression based on the expression of rPrP in inclusion bodies and purification using metal ion affinity column chromatography. Bank vole (BV) rPrP has been shown to be a universal substrate for the amplification of PrP^Sc^ from a variety of different TSEs and host species [12, 13]. Classical sheep scrapie, atypical Nor 98 sheep scrapie, classical BSE, H-type BSE, CWD from elk and deer, hamster, mouse and human samples including sCJD type 1, sCJD type 2, vCJD and iCJD have been detected with BV rPrP in RT-QuIC [14].

Lyophilized protein powders offer advantages over aqueous preparations of protein with regard to storage, shipping, and shelf-life. While there are purification approaches for rPrP available in the literature that include a lyophilization step, the resolubilization step generally involves high concentrations of denaturation and subsequent refolding of the protein [15–18]. Here, we present an approach to prepare lyophilized rPrP suitable for RT-QuIC based detection of PrP^Sc^.

## Main text

### Methods

#### Brain homogenate preparations

Brain homogenates (10% w/v) in 1X PBS (Dulbecco’s PBS, pH 7.4, lacking calcium and magnesium) were prepared from archived tissue available from previously published studies that have been stored at − 80 °C [19, 20].

#### Protein production

*E. coli* (BL21(λDE3)) was transformed with the pET28a vector containing the bank vole *PRNP* gene (amino acids 23–231; GenBank accession number AF367624) and the recombinant bank vole prion protein (BV rPrP) was expressed and purified as previously described for the bovine prion protein with slight modification [21]. Briefly, *E. coli* strains (BL21(λDE3)) containing the bank vole were grown and BV rPrP was expressed in the Overnight Express Autoinduction system (EMD Biosciences). Then cultures were harvested, and the cell pellet (3–4 g) was suspended and lysed to isolate the inclusion bodies. BV rPrP was purified with Ni–NTA (Nickel) resin (Qiagen, #30210) and individual fractions were analyzed by SDS-PAGE. All eluted pooled fractions were dialyzed in 10 mM potassium phosphate (pH 7.0) and lyophilized and stored at − 20 °C. Prior to use, BV rPrP was resolubilized in the double distilled water and used immediately. The concentration of pooled protein eluent or resolubilized lyophilized protein was measured by UV spectroscopy and calculated from the absorbance at 280 nm using an extinction coefficient of 62005 M^−1^ cm^−1^ as calculated for BV protein (23–231) and the final products were compared.

#### RT-QuIC

RT-QuIC reactions were performed as previously described [14, 22–27]. The reaction mix was composed of 10 mM phosphate buffer (pH 7.4), 100 mM to 500 mM NaCl, 0.1 mg/ml freshly prepared or lyophilized and resolubilized BV rPrP, 10 µM thioflavin T (ThT), and 1 mM ethylenediaminetetraacetic acid tetrasodium salt. Aliquots of the reaction mix (98 µL) were loaded into each well of a black 96-well plate with a clear bottom (Nunc, Thermo Fisher Scientific) and seeded with 2 µL of clinical samples, either known scrapie positive or negative control brain homogenate dilutions. The plate was then sealed with plate sealer film and incubated at 42 °C in a BMG FLUOstar Omega plate reader with cycles of 1 min shaking (700 rpm double orbital) and 1 min rest for 100 h. ThT fluorescence measurements (excitation, 460 nm; emission 480 nm, bottom read, 20 flashes per well, manual gain 1400) were taken every 15 min. All reactions for each dilution and each sample were performed in 2 repeats of 4 replicates for a total of 8 RT-QuIC assays. ThT fluorescence data are displayed as the average ThT fluorescence of four technical replicates for each time point and, to be considered positive, the ThT fluorescence of at least two replicate reactions must be positive. As previously described for classification of positive samples by RT-QuIC, the positive threshold was calculated as the mean value of non-inoculated control sheep brain homogenates plus 10 standard deviations [22, 28, 29].

#### Secondary structure and stability evaluation by Far-UV circular dichroism spectroscopy

Far UV circular dichroism (CD) spectra were recorded on a Jasco J-815 spectropolarimeter equipped with a temperature control. Spectra were recorded by averaging three scans in the 200–260 nm range at a scan rate of 10 nm/min. Spectra were acquired at a protein concentration of 2–3 µM of freshly prepared or lyophilized and resolubilized BV protein using 1 cm path length cell to verify if lyophilized PrP is correctly folded compared to freshly prepared BV rPrP. Thermal denaturation curves were monitored by CD at 222 nm over the temperature range of 20–85 °C. The heating rate in all experiments was 1 °C/min. CD signals were plotted as a function of temperature and fit to determine the temperature of unfolding [30].

### Results

#### Protein recovery after lyophilization and resolubilization

**Fig. 1:**
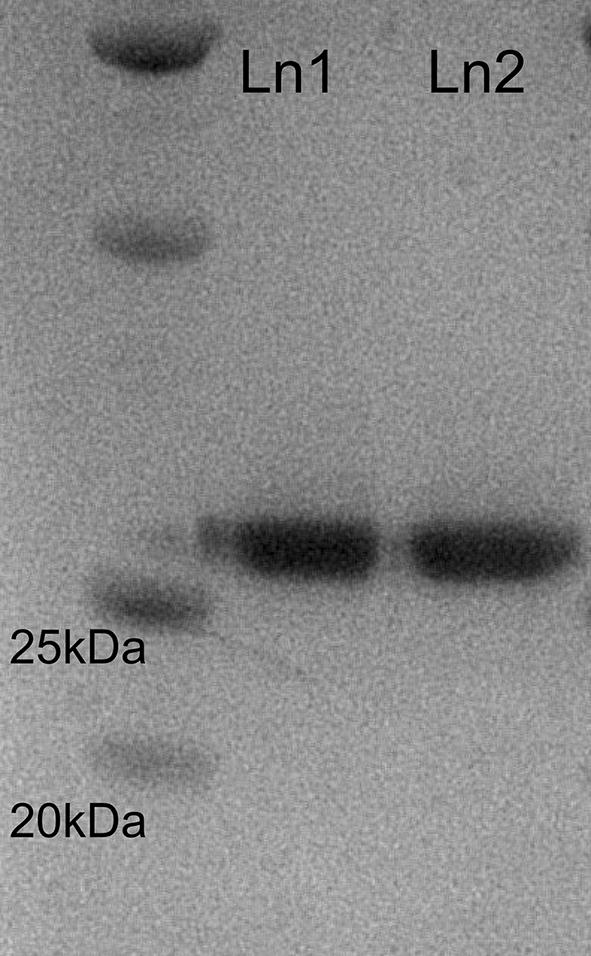
SDS-PAGE for purified BV rPrP. Lane 1: purified BV rPrP protein solution, Lane 2: Lyophilized and resolubilized BV rPrP

In order to investigate protein loss due to the lyophilization and resolubilization, following purification and refolded BV rPrP in 10 mM potassium phosphate pH 7.0 was lyophilized. Following lyophilization the protein was then resolubilized in a volume of distilled water equivalent to the total volume of protein solution prior to lyophilization. Based upon the absorbance at 280 nm and the extinction coefficient of 62005 M^−1^ cm^−1^ it was determined that protein recovery following lyophilization and resolubilization was greater than 95%. As expected, the apparent molecular weight as determined by SDS-PAGE was not affected by the lyophilization process indicating that there was no fragmentation or degradation (Fig. 1).

#### Secondary structure and thermal denaturation of BV rPrP with and without lyophilization

**Fig. 2:**
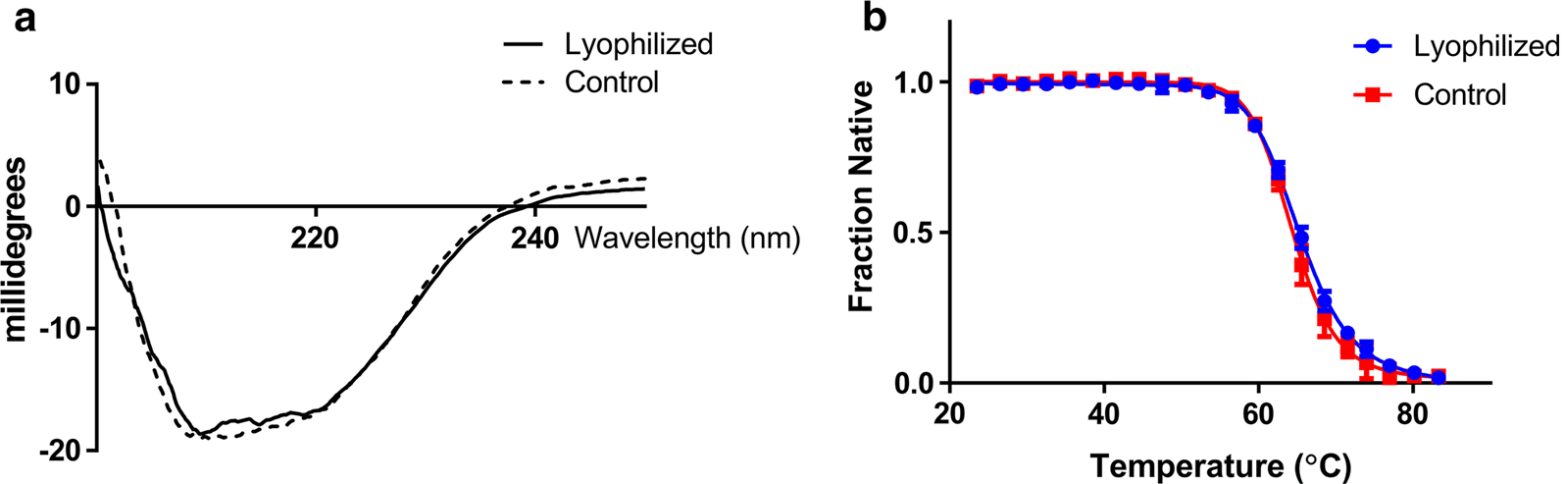
Circular dichroism characterization of freshly prepared and resolubilized BV rPrP. **a** Far-UV circular dichroism spectra of freshly prepared (dotted line) and lyophilized and resolubilized (line) BV rPrP (23–231) and **b** thermal unfolding curves for lyophilized and resolubilized (red) and freshly prepared (blue) BV rPrP in 10 mM phosphate buffer, pH 7.0

In order to investigate whether lyophilization influenced the folding or stability, BV rPrP was analyzed with regard to secondary structure and thermal denaturation. Characteristic minima at 208 nm and 222 nm were observed in the far-UV CD spectra demonstrated with the spectra of the lyophilized BV rPrP essentially indistinguishable to that of the freshly prepared protein (Fig. 2a). In addition, thermal denaturation curves of lyophilized and freshly prepared BV protein were measured to establish any changes in the cooperative folding that resulted from the lyophilization and resolubilization. The thermal unfolding was cooperative for both preparations and the midpoint for BV rPrP was 65.3 ± 0.3 °C with and 64.4 ± 0.5 °C without the lyophilization and resolubilization steps (Fig. 2b).

#### Lyophilized BV rPrP is suitable for RT-QuIC based detection of PrP^Sc^

**Fig. 3:**
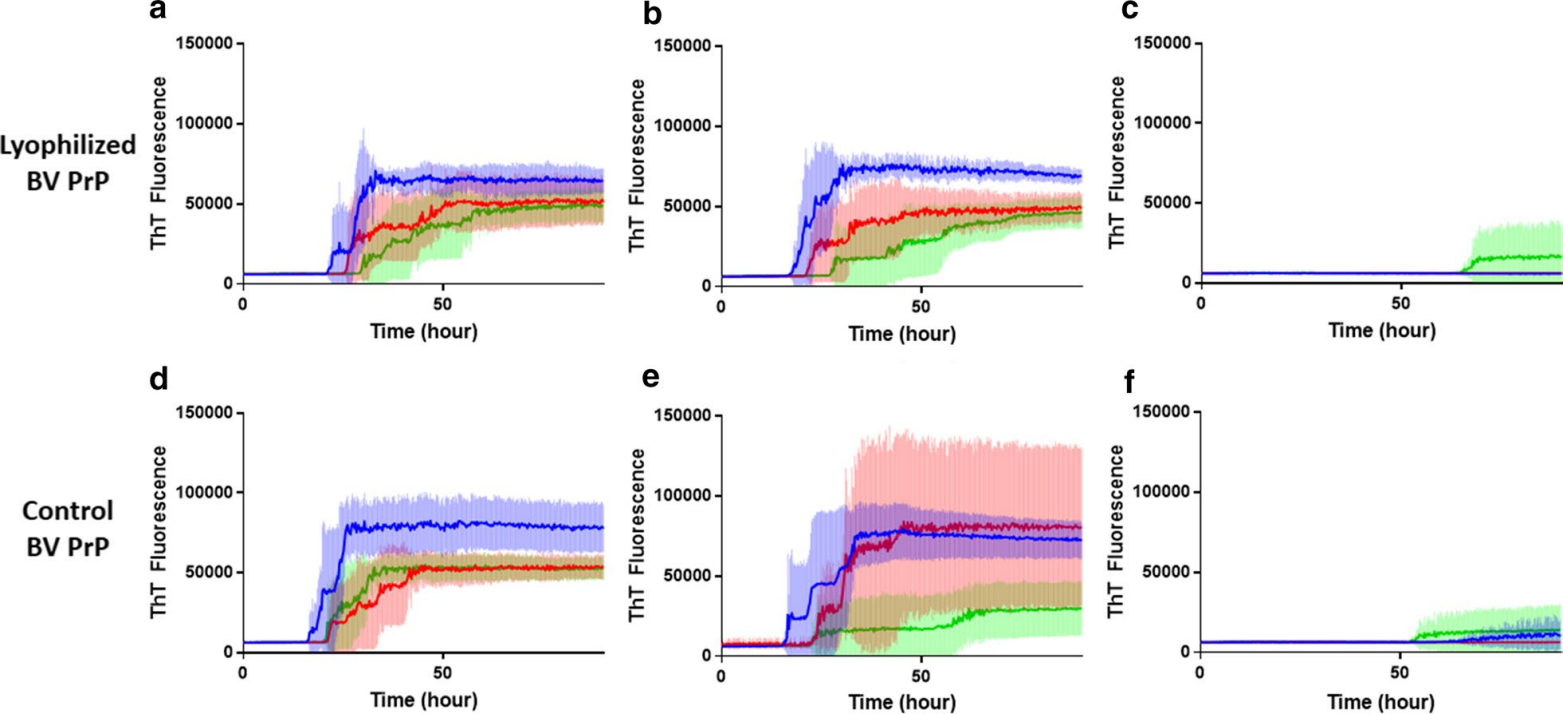
Comparison of seeding activity of RT-QuIC reactions using **a**–**c** resolubilized after lyophilization and **d**–**f** freshly prepared BV rPrP as substrates. RT-QuIC reactions were seeded with 10^−3^ (blue), 10^−4^ (red), and 10^−5^ (green) dilutions of two scrapie-infected sheep brains from 2 different scrapie positive sheep **a** and **d** are the same infected sheep, **b** and **e** are the same infected sheep and a negative control (uninfected sheep, **c** and **f**) brain homogenates with the addition of 0.001% of SDS. Shown are the average ThT fluorescence readings (thick lines) with standard deviations (thin lines) determined from all replicates (four replicate reactions per brain dilution). The results of **a**, **b**, **d** and **e** all meet the criteria to be determined positive by RT-QuIC as defined in the methods

To evaluate whether lyophilized BV protein can be used as a substrate for RT-QuIC reactions, assays containing lyophilized or freshly prepared recombinant BV protein were seeded with dilutions of a 10% (w/v) brain homogenate from 2 different scrapie positive sheep (Fig. 3a, b, d, e) or a confirmed negative control (Fig. 3c, d). Assays utilizing lyophilized BV substrate showed fibril formation with all brain dilutions 10^−3^, 10^−4^, and 10^−5^ as freshly prepared BV substrate showed similar seeding activity for a given animal regardless of whether the substrate was freshly prepared or lyophilized and resolubilized. Neither substrate produced fibril when seeded with brain homogenate from a non-inoculated control based on the absence of an increase in ThT fluorescence (Fig. 3c, f).

### Discussion and conclusions

With this study, we provide proof of concept that lyophilized BV rPrP can be utilized for RT-QuIC reactions based on detection of PrP^Sc^ with lyophilized and resolubilized BV rPrP substrate. Overall, the lyophilization and resolubilization step results in minimal loss in protein, an absence of an appreciable change in secondary structure or temperature of unfolding, and RT-QuIC results of comparable sensitivity to freshly prepared protein.

By no means is this the first use of lyophilization and resolubilization of rPrP. Lyophilized recombinant prion protein has been previously included as part of rPrP preparation protocols, although when included it is typically followed by a resolubilization step including either chemical or thermal unfolding and subsequent refolding prior to use [15–18]. However, this is the first study, to which the authors are aware, that has utilized lyophilized rPrP as a substrate for RT-QuIC.

RT-QuIC has been used for an efficient tool to detect PrP^Sc^ from humans and animals [9–11, 31–34], even in the presymptomatic stage of disease [35–37]. RT-QuIC has also been shown to be potentially useful for studies such as drug prescreening, prion strain discrimination, and screening for prion contamination [38]. A variety of substrates have been applied for RT-QuIC reactions including hamster (23–231 and 90–231) [31], hamster-sheep chimera [39], mouse (23–231) [25], sheep (25–234) [22], human (23–231) [32], deer (24–234) [31], and elk (24–234) [40, 41]. Of the studied PrP sequences, bank vole prion protein appears to be the most practical substrate for diagnostic settings due to its reputation as a universal substrate for RT-QuIC [14]. Also, BV rPrP supports detection of different seeding activity and distinct products of RT-QuIC reactions for different types of human PrP^Sc^ [14].

RT-QuIC has been shown to be sensitive and specific for a number of applications in TSE diagnostics. However, the need for a continued source of freshly prepared and purified rPrP is a limitation of the technique. To address this, researchers have aliquoted large preparations of protein and stored them at − 80 °C with a claimed storage life on the order of 6 months [23]. While the limit of storage lifetime was not established in this study, lyophilized rPrP should extend storage lifetime at temperatures below and above freezing relative to storage in aqueous solution.

In this study, we show that lyophilized BV rPrP can be applied for RT-QuIC reactions to detect PrP^Sc^ in scrapie-infected clinical samples by amplification of fibril content present in a scrapie positive brain homogenate. The effect of lyophilization on BV rPrP was determined to be negligible by comparison to freshly prepared protein solution with regard to secondary protein structure from CD spectra, melting temperature from thermal denaturation curves, and seeding activity from RT-QuIC Across these 3 features we found no significant change as a result of lyophilization. Therefore, lyophilization is a suitable means to preserve BV rPrP for shipping and storage with the ultimate goal of efficient use of RT-QuIC for laboratory research and diagnostic applications.

## Limitations

The data presented here shows that the use of lyophilized BV rPrP is feasible with little or no change to the results found for freshly prepared BV rPrP. However, there are limitations to this study. Namely we did not fully determine the storage shelf life of lyophilized BV rPrP nor did we assess the storage of BV rPrP over a variety of temperatures, rather we limited our evaluation to 4 °C to approximate cold room storage or shipping on wet ice and a time limit consistent with storage of freshly prepared BV rPrP to facilitate direct comparison. While determining the time and temperature limits would undoubtedly be important for development of a distributable test kit this study provides an important proof of principle assessment of the applicability of including lyophilization in the preparation of BV rPrP for the purposes of RT-QuIC, an important step toward development of a distributable test kit.

## Abbreviations

RT-QuIC: real time quaking induced conversion
TSEs: transmissible spongiform encephalopathies
PrP^C^: cellular prion protein
PrP^Sc^: pathogenic prion protein
CWD: chronic wasting disease
CJD: Creutzfeldt–Jakob disease
EIA: enzyme immunoassay
PMCA: protein misfolding cyclic amplification
rPrP: recombinant prion protein
BV: bank vole
sCJD: sporadic CJD
ThT: thioflavin T
CD: circular dichroism
